# Curcumin analogue T83 exhibits potent antitumor activity and induces radiosensitivity through inactivation of Jab1 in nasopharyngeal carcinoma

**DOI:** 10.1186/1471-2407-13-323

**Published:** 2013-07-01

**Authors:** Yunbao Pan, Mengyao Wang, Xianzhang Bu, Yinglin Zuo, Sumei Wang, Dujuan Wang, Qing Liu, Bojin Su, Tao Xu, Chunhua Wang, Francois X Claret, Huiling Yang

**Affiliations:** 1 Department of Pathophysiology, Zhongshan School of Medicine, Sun Yat-Sen University, Guangzhou, Guangdong 510080, People’s Republic of China; 2 Department of Microbial and Biochemical Pharmacy, School of Pharmaceutical Sciences, Sun Yat-sen University, Guangzhou 510006, People's Republic of China; 3 Department of Systems Biology, Unit 950, The University of Texas MD Anderson Cancer Center, 1515 Holcombe Blvd, Houston, TX 77030, USA; 4 Experimental Therapeutic Academic Program and Cancer Biology Program, The University of Texas Graduate School of Biomedical Sciences at Houston, 6767 Bertner Ave, Houston, TX 77030, USA

**Keywords:** Nasopharyngeal carcinoma, Tumorigenesis, Epstein-Barr virus–associated malignancy, Jab1/CSN5, Curcumin

## Abstract

**Background:**

Nasopharyngeal carcinoma (NPC) is an Epstein-Barr virus–associated malignancy that is most common in East Asia, Africa, and Alaska. Radiotherapy is the main treatment option; unfortunately, disease response to concurrent radiotherapy and chemotherapy varies among patients with NPC, and in many cases, NPC becomes resistant to radiotherapy. Our previous studies indicated that Jab1/CSN5 was overexpressed and plays a role in the pathogenesis and radiotherapy resistance in NPC. Therefore, it is important to seek for innovative therapeutics targeting Jab1/CSN5 for NPC. In this study, we explored the antitumor effect of a curcumin analogue T83 in NPC, and found T83 exhibits antitumor activity and induces radiosensitivity through inactivation of Jab1 in NPC.

**Methods:**

NPC cell viability and proliferation were detected by the 3-(4,5-dimethylthiazol-2-yl)-2,5-diphenyltetrazolium bromide (MTT) and colony formation assays. Cell cycle distribution was detected with use of flow cytometry. Apoptosis was examined by using the Annexin V/propidium iodide staining assay and cleavage poly(ADP-ribose polymerase (PARP) and cleavage caspase-3 expression. Jab1 expression was examined by Western blotting.

**Results:**

A growth inhibitory effect was observed with T83 treatment in a dose- and time-dependent manner. T83 significantly induced G2/M arrest and apoptosis in NPC. In addition, T83 inhibited Jab1 expression and sensitized NPC cells to radiotherapy.

**Conclusion:**

Our data indicate that T83 exhibits potent inhibitory activity in NPC cells and induces radiotherapy sensitivity. Thus, T83 has translational potential as a chemopreventive or therapeutic agent for NPC.

## Background

Human nasopharyngeal carcinoma (NPC) is one of the most common cancers in Southern China [1], and the highest incidence of this disease (peaking at 50 per 100,000 persons per year) is found in this area, especially among those of Cantonese origin [2]. Epstein-Barr virus infection, environmental factors, and genetic susceptibility are all associated with NPC [3,4]. Although NPC is a relatively radiosensitive disease, the therapeutic effect of radiotherapy is not satisfactory because of radioresistance [5]. To determine the mechanism of radioresistance in this disease, our research group established the radioresistant NPC cell line (CNE2R) based on CNE2 [6]. Genetic alterations have been reported in NPC, and our recent findings showed that one such alteration, c-Jun activation domain-binding protein-1/constitutive photomorphogenic-9 signalosome (Jab1/CSN5), which negatively regulates p27, is overexpressed in this disease [7] and thus contributes to NPC resistance to radiotherapy and chemotherapy [8,9]. However, specific inhibitors targeting Jab1 are largely undetermined.

Curcumin is a well-known chemopreventive agent that has potent anticancer activity in a wide variety of tumor cells [10,11]. Unfortunately, many preclinical and clinical studies have indicated poor bioavailability and rapid metabolism of curcumin, due to its instability under certain physiologic conditions, which have limited its application in anticancer therapy [12]. Consequently, analogues of curcumin with similar safety profiles but with increased anticancer activity have been developed in recent years [11,13-15]. Interestingly, Jun Li et al. reported that PEGylated curcumin inhibits pancreatic cancer cell proliferation through suppression of Jab1/CSN activity [16]. Recently, our group synthesized a series of new 4-arylidene curcumin analogues that can effectively inhibit proliferation of cells in a panel of human cancer cell lines such as CNE2, SW480, MCF-7, A549, and HepG2 [17-19].

Our findings indicate that a new 4-arylidene curcumin analogue, T83, through inhibition of Jab1, exerts an antitumor effect in CNE2 and CNE2R cells by reducing cell growth, arresting the cell cycle, and increasing apoptosis. Furthermore, T83 sensitizes NPC to radiotherapy. This work identifies T83 as a potential targeted therapy that sensitizes cells prior to conventional radiotherapy, thus providing more effective treatment for NPC patients.

## Methods

### Materials

Cell culture reagents and fetal bovine serum (FBS) were obtained from Invitrogen (Carlsbad, CA, USA). The antibodies used were poly(ADP-ribose polymerase (PARP),p53 and p27 (BD Biosciences PharMingen, San Diego, CA, USA), caspase-3 (Cell Signaling Technology, Beverly, MA, USA), Jab1 (Santa Cruz, CA, USA), and β-actin (Sigma-Aldrich, St. Louis, MO, USA). Oligofectamine reagent was from Invitrogen, the Western Lightning Chemiluminescence Plus reagent was from Thermo Scientific Pierce (Rockford, IL, USA), MTT and DMSO were from sigma (St. Louis, MO, USA). The Annexin-V/propidium iodide (PI) kit was from BD Biosciences (Palo Alto, CA, USA). T83, which was synthesized in our laboratory, was dissolved in dimethyl sulfoxide (DMSO) to prepare a 10 mM stock solution and stored at −20°C.

### Synthesis of T83

The procedure used for the synthesis of T83 was described previously [19]. Generally, an amount of 1.0 mmol of 1, 3-diketones curcumin analogs [20] and 2 mmol of the corresponding benzaldehyde as well as 25 mL toluene were added to a three-neck rounded flask equipped with a water dispenser. Pyridine (4.0 mg, 0.05 mmol, in 0.1 mL toluene) and acetic acid (4.8 mg, 0.08 mmol, in 0.1 mL toluene) were added as catalysts. The reaction mixture was stirred in 140°C overnight, and the generated water was removed by water dispenser during the whole reaction. Then the reaction mixture was washed with water (10 mL, twice) to remove pyridine and acetic acid. Next the organic layer was evaporated under vacuum to get raw product. Finally the product was purified by using silica gel column chromatography.

### Cell lines and culture conditions

The human NPC cell lines CNE1(well-differentiated) and CNE2 (poorly differentiated) were obtained from the Experimental Animal Center of Sun Yat-Sen University. The radioresistant NPC cell line CNE2R was generated from parental CNE2 cells as previously described [6] and identified by analysis of DNA microsatellite short tandem repeats (STR) (see additional file 1). The NPC cells were cultured in RPMI-1640 medium with 10% FBS and antibiotics (100 U/mL of penicillin and 100 μg/mL of streptomycin) in cell culture incubators at 37°C and aired with 5% CO_2_. Cells were γ-irradiated with use of a JL Shepherd and Associates (San Fernando, CA, USA) Mark I-30 ^137^Cs irradiator at MD Anderson Cancer Center.

### MTT cell viability assay

The 3-(4,5-dimethylthiazol-2-yl)-2,5-diphenyltetrazolium bromide (MTT) assay was used to evaluate cell viability, as described previously [8]. Briefly, NPC cells were seeded in 96-well plates (2000 cells/well) in RPMI-1640 medium with 10% FBS. The following day, the cells were treated with indicated concentration of T83 or curcumin and incubated for 48 h. A total of 20 μL of MTT (5 mg/mL) was added to each well and incubated for 3.5 h. The medium was discarded, and 150 μL of DMSO was added to each well and incubated for 10 min. The absorbance was read at 570 nm. The concentration of drug required to obtain 50% maximal inhibition in cell viability was indicated by IC_50_.

### Colony formation assay

We performed the colony formation assay as previously described [8]. Generally, the NPC cells (200 cells/well) were plated in six-well plate for growth analysis in RPMI-1640 medium with 10% FBS. The following day, the cells were treated with indicated doses of T83. The NPC cells were grown at 37°C for 12 days. The effect of the drugs on growth was determined by colony growth. Colonies were stained with 0.1% crystal violet and scored by counting with an inverted microscope, using the standard definition of a colony consisting of 50 or more cells. Numbers were normalized as a percentage of colonies formed in DMSO treatment.

### Measurement of cell cycle and apoptosis by flow cytometry

The treated cells were collected and fixed overnight in 75% cold ethanol at 4°C. Cells were then washed twice in cold phosphate-buffered saline and labeled with PI (Sigma-Aldrich), as previously described [8], and analyzed immediately after staining with use of an Epics Elite flow cytometer (Coulter Corporation, Miami, FL, USA) and WinMDI29 software.

Flow cytometric analysis, described previously [8], was used to differentiate between living, early apoptotic, late apoptotic/necrotic, and necrotic cells by staining with Annexin V and PI. Briefly, after the indicated treatment, all of the cells were collected and resuspended in 100 μL of binding buffer containing Annexin V and PI, according to the manufacturer’s recommendations. Quantification of Annexin V and PI binding was performed by a FACScan flow cytometer (Becton-Dickinson).

### Small interfering RNA transfection

The negative control small interfering RNA (siRNA; siControl 3) and siRNA targeting human Jab1/CSN5 (siCOPS5 #18546) were purchased from Ambion, Inc. (Austin, TX, USA). Transient transfections of NPC cells were performed as described previously [8] by using the Oligofectamine (Invitrogen) protocol with 5 nmol siRNA in RPMI-1640 with 10% FBS and no penicillin-streptomycin.

### Cell extracts and immunoblotting

Cells in the log phase of growth were collected, washed twice in cold phosphate-buffered saline, and lysed as described previously [7]. Proteins in the total cell lysates were separated by 10% sodium dodecyl sulfate–polyacrylamide gel electrophoresis (SDS-PAGE), transferred to nitrocellulose membranes, and probed with anti-Jab1,anti-p53, anti-p27, anti-PARP, and anti-caspase-3. actin was used as the internal positive control for all immunoblots. Immunoreactive bands were detected with use of horseradish peroxidase–conjugated secondary antibodies with the Western Lightning Chemiluminescence Plus reagent. Protein levels were quantified with use of ImageJ software (National Institute of Mental Health, Bethesda, MD, USA; http://rsb.info.nih.gov/ij). Activities of PARP and caspase-3 were measured as the proportion of cleavage band intensity to the total bands and were calculated as follows: % PARP or caspase-3 = 100% × Tc/Tt, where Tc is the intensity value of the cleavage bands and Tt is the intensity value of total bands.

### Statistical analysis

Statistical analysis of the results was performed by using the Student’s *t* test for only two groups or by using one-way analysis of variance for more than two groups. Differences between groups were considered statistically significant at *P* < 0.05. All computations were performed with SPSS 19.0 (SPSS, Chicago, IL, USA).

## Results

### T83 inhibited cell viability

**Figure 1:**
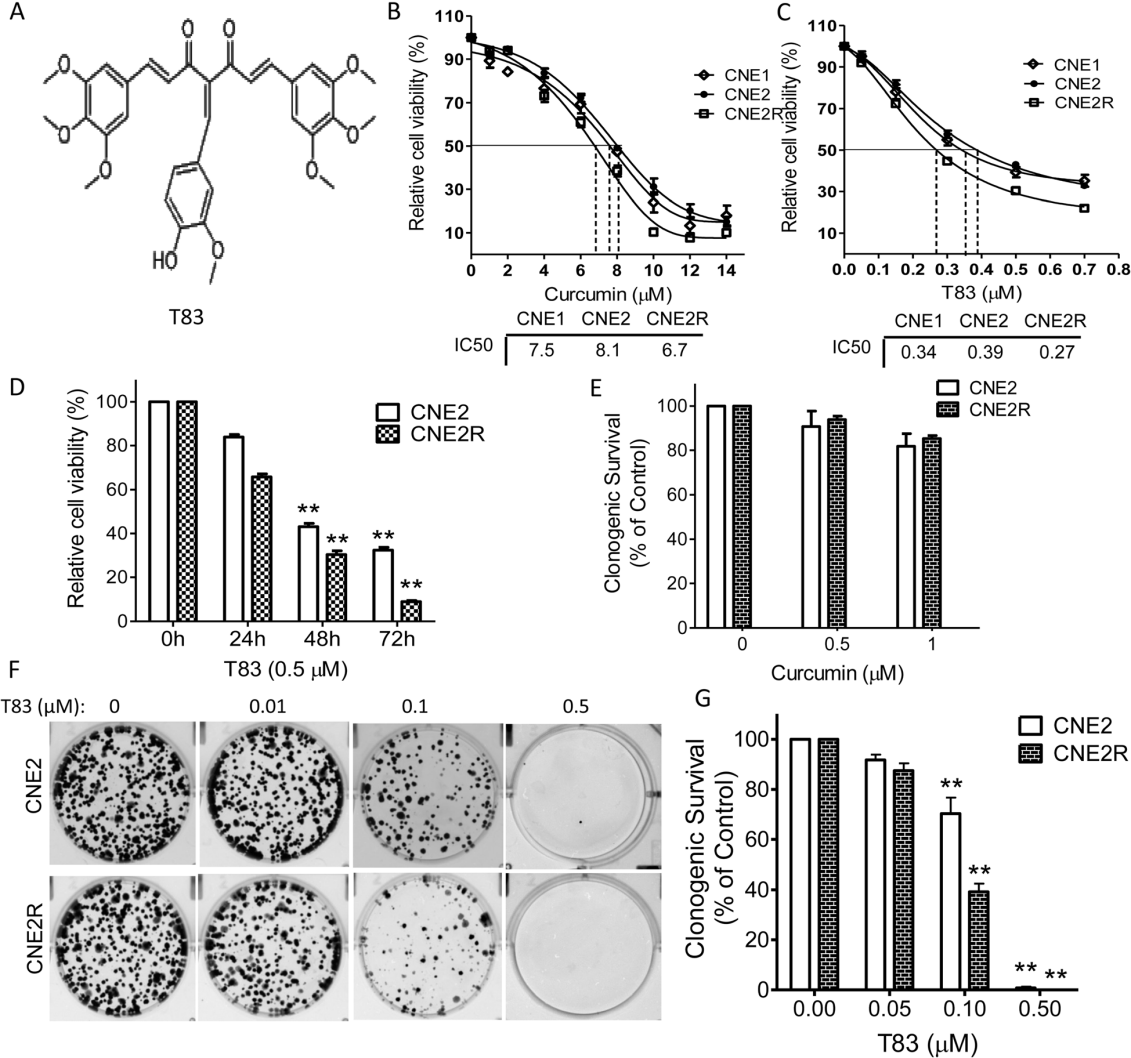
**T83 inhibited viability of NPC cells.** Chemical structures of T83 **(A)**. NPC cells were incubated with various concentrations of curcumin **(B)** or T83 **(C)** for 48 h or exposed to 0.5 μM T83 **(D)** for 24, 48, and 72 h; cell viability was then quantified by the MTT assay. **(E)** Clonogenic survival of NPC cells treated with curcumin. **(F)** Representative results of colony formation assays with CNE2 and CNE2R cells. **(G)** Quantification of colonies. All data represent three independent experiments, mean ± s.d. ^*^P < 0.05, ^**^P < 0.01.

We first examined the cytotoxicity of T83 by assessing CNE1, CNE2 and CNE2R cells. We exposed cells from NPC cell lines to various concentrations of T83. In our studies, T83 showed growth-suppressive activity in the NPC cell lines tested in a dose- and time-dependent manner (Figure 1C and 1D). However, T83 exhibited greater inhibition than curcumin. IC_50_ values of T83 were 0.34 μM for CNE1, 0.39 μM for CNE2 and 0.27 μM for CNE2R, respectively, which are substantially more potent than curcumin (IC_50_ values 7.5 μM, 8.1 μM and 6.7 μM) (Figure 1B).. In addition, we performed a colony formation assay to determine the effect of T83 on NPC cells’ proliferation. As expected, T83 significantly inhibited colony formation, with more than 99% inhibition at 0.5 μM treatment in the cell lines tested (Figure 1F and G), while the survival rated reduced only 10% (CNE2) and 7% (CNE2R) at the same concentration of curcumin treatment (Figure 1E). These results demonstrated that T83 was more potent than curcumin in inhibiting cell viability and proliferation in NPC cells.

### T83 induced cell cycle arrest and apoptosis in NPC

**Figure 2:**
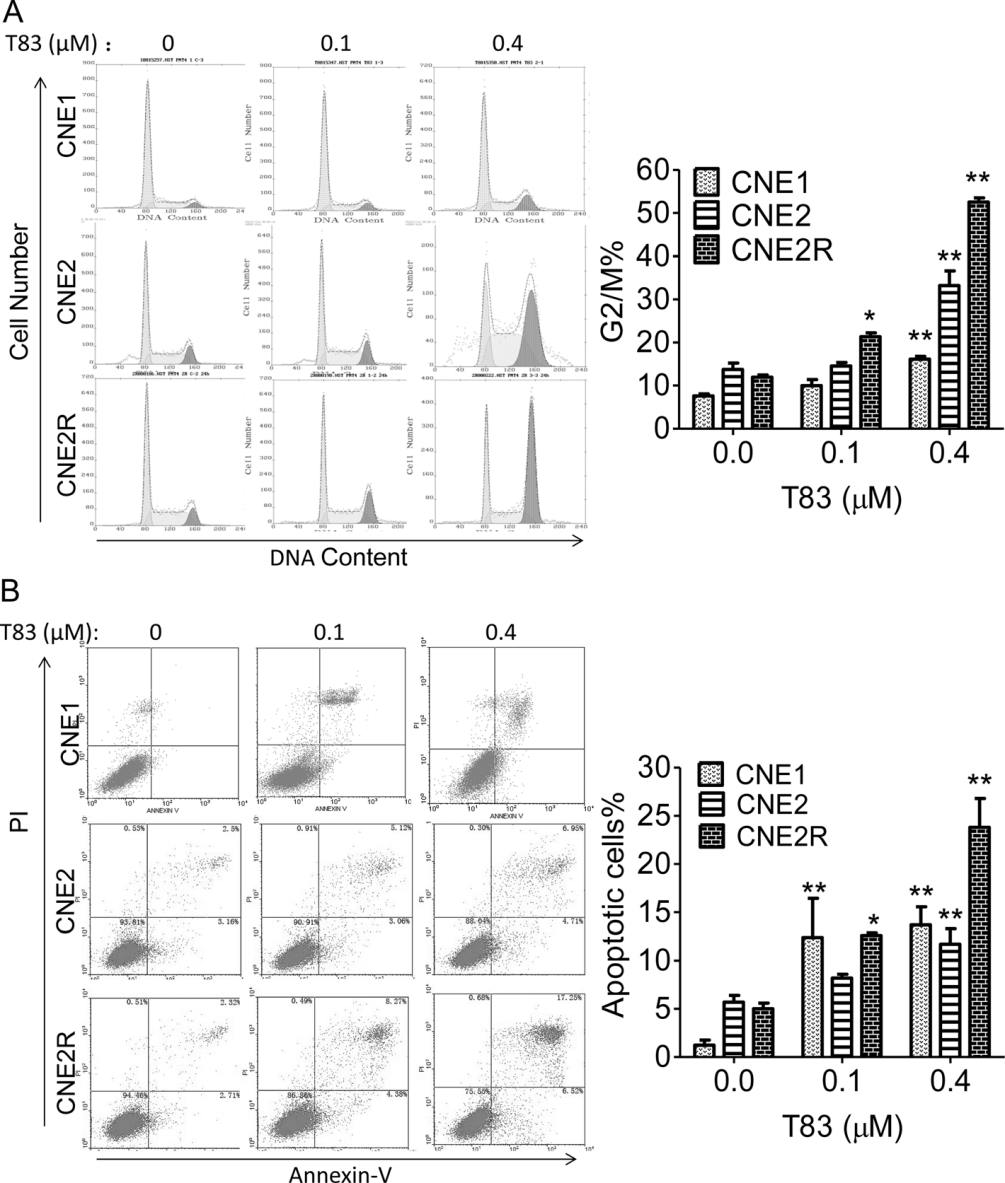
**T83 induced cell cycle arrest and apoptosis in NPC cells. (A)** The effects of T83 on cell cycle distribution in NPC cells were determined by flow cytometric analysis. Left, NPC cells were treated with DMSO control (0) or indicated concentration of T83 for 24 h and then fixed in ethanol and stained with propidium iodide. Right, Quantification of G2/M phase. **(B)** NPC cells were treated with DMSO control (0) or indicated concentration of T83 for 48 h and then stained with Annexin-V/PI and detected by flow cytometry. Right, Quantification of cell apoptosis. All data represent three independent experiments, mean ± s.d. ^*^P < 0.05, ^**^P < 0.01.

Consistent with our observations in NPC cells, flow cytometric analysis revealed that T83 induced cell cycle arrest in CNE1 cells at the G2/M phase, with the percentage of G2/M cells changing from 7.5% in DMSO-treated controls to 16.1% with 0.4 μM T83 treatment for 24 h. For the CNE2 and CNE2R cells, we got similar results: the proportion of G2/M phase cells changed from 13.7% to 33.2% in CNE2 cells and from 11.9% to 52.5% in CNE2R cells (Figure 2A).

We next determined whether T83-induced cell viability inhibition is followed by increased apoptosis. NPC cells were treated with T83 for 48 h and analyzed by Annexin-V and PI staining, which detects apoptosis. Treatment of NPC cells with T83 resulted in 10 times more apoptotic CNE1 cells, 2 times more CNE2 cells and 4.8 times more apoptotic CNE2R cells (Figure 2B).

### Inactivation of Jab1 by T83 is dose- and time-dependent

**Figure 3:**
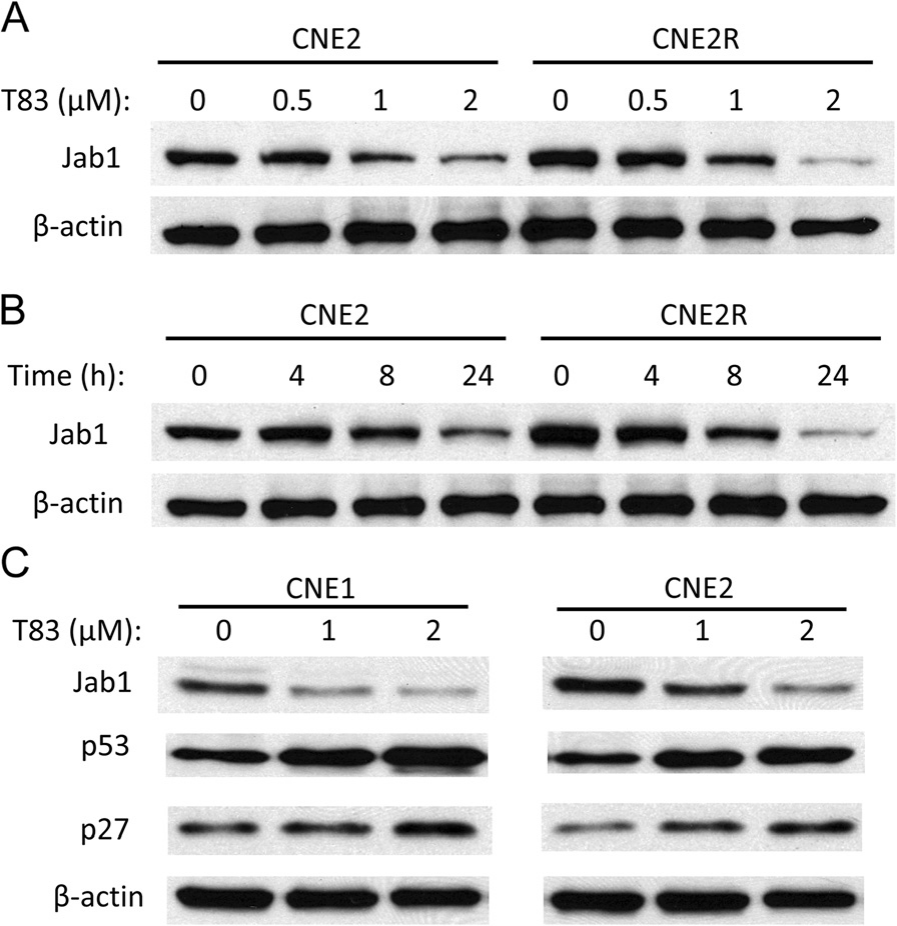
**T83 inhibited Jab1 in a dose- and time-dependent manner in NPC cells.** Cells from NPC cell lines were treated with and without T83 at the specified concentrations for 24 h **(A)** or exposed to 2 μM T83 for various time points **(B)**. Expression of Jab1,p53, p27 and β-actin were examined by Western blotting **(C)**. DMSO was used as control in “0” groups.

To further determine the effect of T83 on Jab1 inactivation in NPC, we exposed CNE2 and CNE2R cells to various concentrations of T83. As shown in Figure 3A and 3B, T83 inhibited Jab1 activation in a dose- and time-dependent manner. As Jab1 can promote the degradation of p27 [7] and p53 [21], we further explored the effect of T83 on these two proteins. As expected, we found that the decrease of Jab1 induced by T83 treatment was associated with an increase of p27 and p53 (Figure 3C). These data suggest that T83 inhibits Jab1 activity in NPC.

### Jab1 expression was associated with T83 efficacy

**Figure 4:**
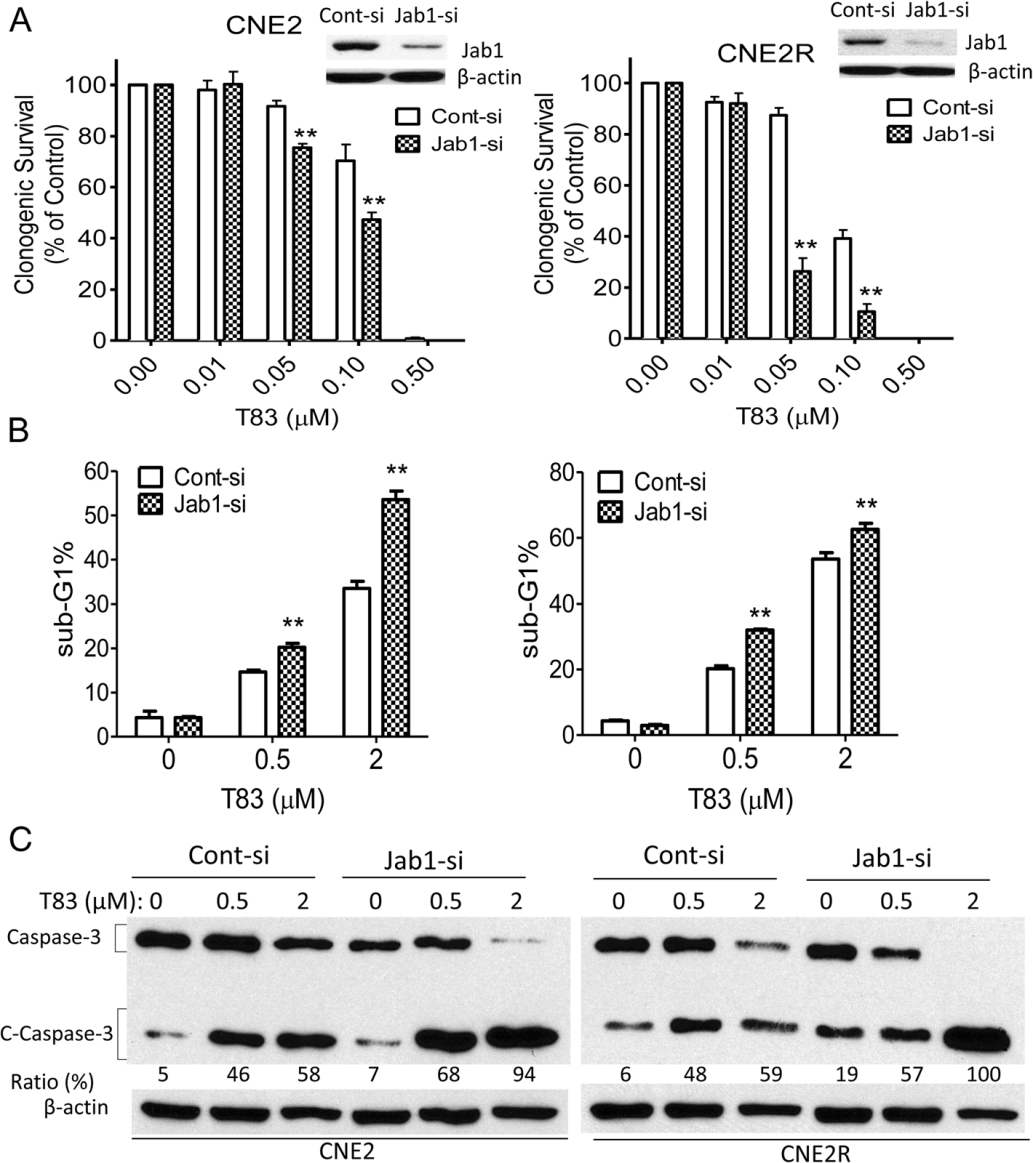
**Jab1 knockdown enhanced the effects of T83 on cell viability inhibition and apoptosis induction.** CNE2 cells (left) and CNE2R cells (right) were transfected with control siRNA (Cont-si) or Jab1 siRNA (Jab1-si) and then exposed to the indicated doses of T83 for 48 h; they were then examined for colony formation **(A)**, sub-G1 **(B)**, and cleaved caspase-3 (C-Caspase 3) **(C)**. Protein levels were quantified with use of ImageJ software. All data represent three independent experiments, mean ± s.d. DMSO was used as control in “0” groups.

To further explore the role of Jab1 in T83 action, we sought to determine whether downregulation of Jab1 would influence T83 efficacy. Thus, NPC cells were transfected with Jab1 siRNA, and cell survival was measured by the colony formation assay. Jab1 knockdown of CNE2 cells resulted in increased T83-induced cell inhibition, with 23% and 17% higher cell survival inhibition than that seen in control cells transfected with a vector at 0.05 and 0.1 μM T83, respectively (Figure 4C, left). Similar results were seen in CNE2R cells transfected with Jab1 siRNA, which displayed increased T83-induced cell inhibition, with 61% and 29% higher cell survival inhibition than that seen in control cells transfected with a vector at 0.05 and 0.1 μM T83, respectively (Figure 4C, right).

Consistent with the observations described above, CNE2 cells with reduced Jab1 expression showed increased efficacy of T83 (0.5 and 2 μM) compared with control cells transfected with scrambled siRNA; the hypodiploid (sub-G1) DNA content, reflecting apoptosis, was increased by 78% and 36%, respectively, at these concentrations (Figure 4B, left). The CNE2R cells behaved similarly: cells in the sub-G1 phase increased by 60% and 19%, respectively (Figure 4B, right) at the above concentrations of T83. Similar results were seen when we tested caspase-3 cleavage. CNE2 cells (Figure 4C, left) and CNE2R cells (Figure 4C, right) transfected with Jab1 siRNA displayed increased T83-induced caspase-3 cleavage compared with control cells. Considering our findings together, we conclude that Jab1 levels were associated with T83 efficacy.

### T83 sensitizes NPC cells to radiotherapy

**Figure 5:**
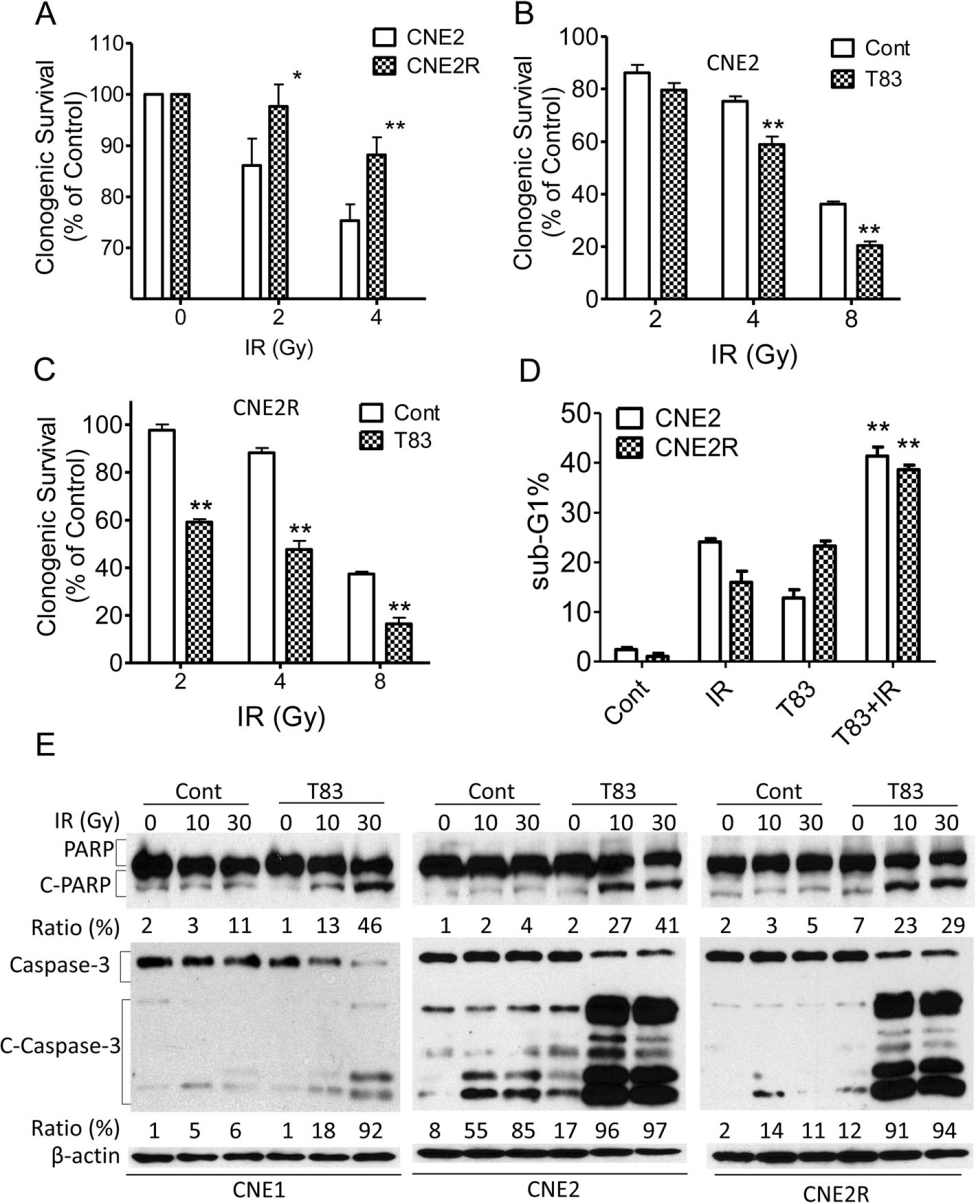
**T83 sensitized NPC cells to radiotherapy.** NPC cells were treated with IR alone or together with 0.05 μM T83 for 48 h and were then examined for colony formation **(A) (B) (C)**, PI staining **(D)**, and cleaved PARP (C-PARP) and caspase-3 (C-Caspase-3) **(E)**. DMSO was used as control in “Cont” groups.

Because radiotherapy is the main treatment for NPC, we investigated whether T83 is involved in the antitumor effects of radiotherapy. We first used the colony formation assay to verify the effects of radiotherapy on CNE2 and CNE2R cells. We observed results similar to those previously reported [6], that CNE2R cells are more resistant to irradiation (IR) (Figure 4A). We next used a suboptimal dose (≤IC_10_) of T83 to examine whether T83 increased the sensitivity of NPC cells to IR. As expected, NPC cells treated with T83 showed increased sensitivity to IR compared with control cells treated with IR alone. The survival rates of CNE2 cells treated with T83 were reduced by 20% and 44% when exposed to 4 and 8 Gy of radiation, respectively (Figure 5B), whereas survival rates of CNE2R cells treated with T83 were reduced by 47% and 57%, respectively, at these exposures (Figure 5C).

We further examined whether T83 could enhance IR-induced apoptosis in NPC cells. We found that IR treatment increased apoptosis in T83-treated cells more than in control cells, i.e., by 70% in CNE2 cells and by 140% in CNE2R cells, as measured by PI staining (Figure 5D). Proteolytic cleavage of PARP and cleaved caspase-3 are the hallmarks of apoptosis [22,23]. Thus, we also examined the effects of T83 on the proteolytic cleavage of PARP and cleaved caspase-3 in response to IR. Compared with results for the control cells, IR consistently induced more proteolytic cleavage of PARP (35% change in CNE1, 37% change in CNE2 cells, 24% change in CNE2R cells) and cleaved caspase-3 (86% change in CNE1, 41% change in CNE2 cells, 83% change in CNE2R cells) in T83-treated cells (Figure 5D).

## Discussion

In the current study, we have presented evidence showing the effective inhibition of Jab1 by the new 4-arylidene curcumin analogue, T83, which resulted in antitumor effects in NPC cells. These findings suggest that T83 may be effective in suppressing NPC cell growth in patients with cancer.

We first confirmed that a synthetic analogue of curcumin, T83, could significantly inhibit cell proliferation in NPC cells. T83 is highly effective in inhibiting cell viability and clone formation in NPC cell lines, and its IC_50_ value is much lower than the IC_50_ values of other curcumin analogues [17]. Furthermore, we have shown that T83 can arrest the cell cycle at the G2/M phase and induce apoptosis in a dose-dependent manner. Previous studies had shown that cells in the G2/M phase are the most sensitive to radiation and that tumor cells in this phase are the most responsive to IR and chemotherapy [24,25]. In our study, the number of radioresistant CNE2R cells in the G2/M phase increased significantly in a dose-dependent way. All of these data supported T83’s effect on reversing radioresistance by increasing G2/M phase accumulation.

Because Jab1 is overexpressed in many cancers including NPC [7], suppression of Jab1 contributes to the sensitivity of cisplatin chemotherapy and radiotherapy [8]. Inhibiting the Jab1 signaling pathway may be an effective strategy in the treatment of NPC, and we here presented the first evidence of T83 activity in NPC. We also found that T83 is effective in inhibiting Jab1 expression in a dose- and time-dependent manner. Given these findings, we examined the potential effects of Jab1 on T83’s activity in NPC. Knockdown of Jab1 enhanced T83’s activity against NPC cells.

Interestingly, all of our experiments showed that radiation-resistant cells (CNE2R) are more sensitive to T83 than the parental CNE2 cells; we therefore speculated that T83 possibly sensitizes NPC cells to radiotherapy. To test this hypothesis, we selected a suboptimal dose (≤IC_10_) of T83 to examine whether T83 increased the sensitivity of NPC cells to IR. Our study suggested that T83 treatment in combination with radiotherapy improve the response of NPC to radiotherapy.

## Conclusions

Taken together, our results indicated that T83 exhibits enhanced antitumor activity in NPC cells. Our findings, which demonstrated that T83 inhibited Jab1 and supported T83 as an anticancer agent, could have important clinical relevance, namely, that administration of T83 could become part of an effective therapeutic regimen for NPC.

## Abbreviations

DMSO: Dimethyl sulfoxide; FBS: Fetal bovine serum; IC50: Concentration of drug required to obtain 50% maximal inhibition in cell viability; IR: Ionizing radiation; MTT: 3-(4,5-dimethylthiazol-2-yl)-2,5-diphenyltetrazolium bromide; NPC: Nasopharyngeal carcinoma; PI: Propidium iodide; siRNA: small interfering RNA.

## Competing interests

The authors declare that they have no competing interests.

## Authors’ contributions

YP conceived of the study, designed and performed the experiments, analyzed data and drafted the manuscript. MW designed and performed the experiments. XB and YZ contributed reagents and performed experiments. SW, DW, QL, BS, TX and CW performed experiments. FXC and HY conceived of the study, and participated in its coordination and helped to draft the manuscript. All authors read and approved the final manuscript.

## Authors’ information

Yunbao Pan and Mengyao Wang co-first author.

## Pre-publication history

The pre-publication history for this paper can be accessed here:

http://www.biomedcentral.com/1471-2407/13/323/prepub

## Supplementary Material

Additional file 1 STR profiling of CNE2 and CNE2R. Click here for file
