# Supplementary figures and images for: Curcumin analogue T83 exhibits potent antitumor activity and induces radiosensitivity through inactivation of Jab1 in nasopharyngeal carcinoma
Source: BMC Cancer. 2013 Jul 1;13:323. doi: 10.1186/1471-2407-13-323 (PMC3706359; doi:10.1186/1471-2407-13-323)

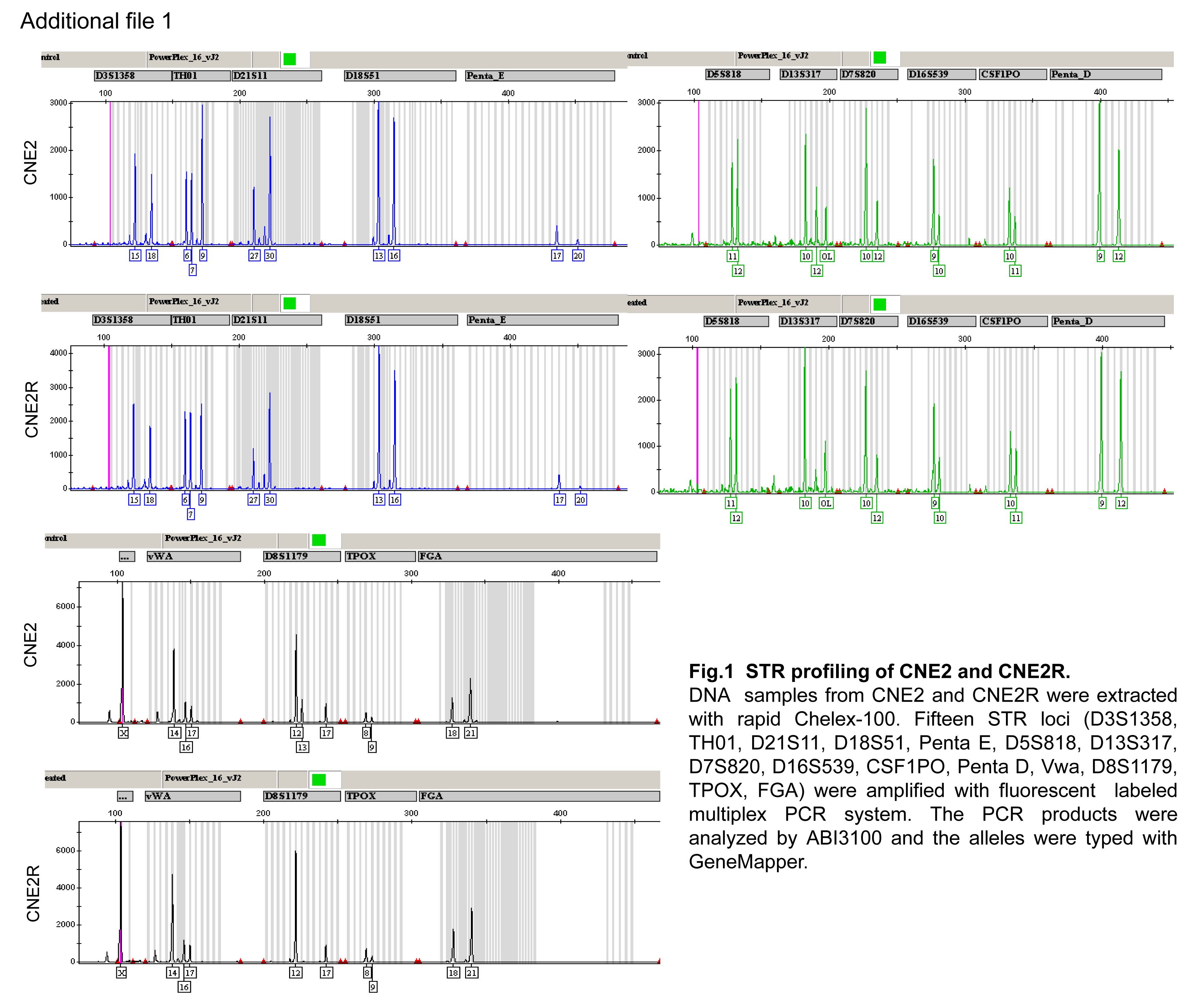

Supplement: Additional file 1 — STR profiling of CNE2 and CNE2R. [file 1471-2407-13-323-S1.tiff]
